# A combined *DPA1∼DPB1* amino acid epitope is the primary unit of selection on the HLA-DP heterodimer

**DOI:** 10.1007/s00251-012-0615-3

**Published:** 2012-04-13

**Authors:** Jill A. Hollenbach, Abeer Madbouly, Loren Gragert, Cynthia Vierra-Green, Susan Flesch, Stephen Spellman, Ann Begovich, Harriet Noreen, Elizabeth Trachtenberg, Tom Williams, Neng Yu, Bronwen Shaw, Katharina Fleischhauer, Marcelo Fernandez-Vina, Martin Maiers

**Affiliations:** 1 National Marrow Donor Program, Minneapolis, MN USA; 2 Children’s Hospital Oakland Research Institute, Oakland, CA USA; 3 Center for International Blood and Marrow Transplant Research, Minneapolis, MN USA; 4 Roche Diagnostics, Pleasanton, CA USA; 5 Fairview Hospital, Minneapolis, MN USA; 6 University of New Mexico, Albuquerque, NM USA; 7 American Red Cross, Dedham, MA USA; 8 Anthony Nolan Trust, London, UK; 9 Haemato-Oncology Research Unit, Division of Molecular Pathology, The Institute of Cancer Research, London, UK; 10 Unit of Molecular and Functional Immunogenetics, Division of Regenerative Medicine, Stem Cells and Gene Therapy, San Raffaele Scientific Institute, Milan, Italy; 11 Stanford University School of Medicine, Palo Alto, CA USA

**Keywords:** DPA1, DPB1, Heterodimer, Haplotype, Amino acid, Epitope

## Abstract

Here, we present results for *DPA1* and *DPB1* four-digit allele-level typing in a large (*n* = 5,944) sample of unrelated European American stem cell donors previously characterized for other class I and class II loci. Examination of genetic data for both chains of the DP heterodimer in the largest cohort to date, at the amino acid epitope, allele, genotype, and haplotype level, allows new insights into the functional units of selection and association for the DP heterodimer. The data in this study suggest that for the *DPA1-DPB1* heterodimer, the unit of selection is the combined amino acid epitope contributed by both the *DPA1* and *DPB1* genes, rather than the allele, and that patterns of LD are driven primarily by dimer stability and conformation of the P1 pocket. This may help explain the differential pattern of allele frequency distribution observed for this locus relative to the other class II loci. These findings further support the notion that allele-level associations in disease and transplantation may not be the most important unit of analysis, and that they should be considered instead in the molecular context.

**Electronic supplementary material:**

The online version of this article (doi:10.1007/s00251-012-0615-3) contains supplementary material, which is available to authorized users.

## Introduction

The human leukocyte antigen (HLA) complex on chromosome 6 is the most polymorphic region of the human genome, and while doubly polymorphic heterodimeric molecules are relatively rare in human biology, this is a defining feature of the HLA class II gene products. Within the class II region, the *DRB1* locus is recognized as significantly more polymorphic relative to the other beta chain genes, with >1,000 alleles recognized to date, and is nearly 150-fold more diverse than the *DRA* locus, where only seven alleles have been identified (http://www.ebi.ac.uk/imgt/hla/stats.html). In contrast, the HLA-DQ and HLA-DP systems are characterized by having more moderate variation overall relative to HLA-DR, but more diversity in genes for the alpha chains, resulting in less imbalance between levels of alpha and beta diversity; the ratio of beta/alpha known alleles is approximately 4:1 for these genes. In the DQ system, alpha chain diversity, coupled with relatively even allele frequency distributions for both *DQA1* and *DQB1* in most human populations (Fernandez-Vina et al. 1991; Solberg et al. 2008; Begovich et al. 1992; Slatkin 2000), potentially increases polymorphism at the heterodimer level. At the same time, the DQ system is characterized by near complete linkage disequilibrium (LD), and evidence suggests that the LD is driven in part by structurally permissive and nonpermissive pairings of the alpha and beta chains, which may restrict overall heterodimer polymorphism. Much previous work (Begovich et al. 1992; Bugawan et al. 2000; Fernandez-Vina et al. 1991; Hollenbach et al. 2001; Klitz et al. 2003) has demonstrated that certain combinations of *DQA1* and *DQB1* alleles are almost never seen on the same haplotype, and there is evidence to suggest that these pairings do not produce stable cell surface heterodimers (Kwok et al. 1993; Kwok and Nepom 1989). In contrast to the balanced polymorphism observed for *DQA1* and *DQB1*, only a few alleles predominate in most human populations for both *DPA1* and *DPB1* (Gendzekhadze et al. 2004; Steiner et al. 2000) (Begovich et al. 2001; Pérez-Miranda et al. 2004; Solberg et al. 2008), resulting in significantly less potential heterodimeric diversity within a given population. However, while much is known about alpha–beta haplotypic associations in the HLA-DQ system, because *DPA1* genotyping is infrequently performed, there have been only limited studies with relatively small sample sizes describing *DPA1∼DPB1* haplotypes (Begovich et al. 2001; Gendzekhadze et al. 2004).

Evidence for lower levels of cell surface expression of the DP molecule relative to other classical HLA molecules (Edwards et al. 1986; Guardiola and Maffei 1993) has historically led to the notion that DP may be less important than other HLA in human health. Furthermore, a lack of evidence for balancing selection, a hallmark of the other class II loci, has been interpreted as an indication that DP may not play an important role in protection from pathogens in humans. In both solid organ and hematopoietic stem cell transplantation (HSCT), standard protocols do not call for matching at DP, and DP typing is not routinely performed. However, recent data suggest that DP match status may play an important role in HSCT outcome (Crocchiolo et al. 2009; Fleischhauer et al. 2006; Gallardo et al. 2004; Shaw et al. 2003, 2006; Vrana et al. 2006; Zino et al. 2004, 2007; Fleischhauer et al. 2012), although the nature and direction of the impact are still the subject of debate. DP molecules can serve as targets of alloreactivity with clinical consequences in HSCT, demonstrated by data showing association of HLA-DP mismatching with relapse, GvHD, rejection and nonrelapse mortality after unrelated HSCT, as well as data suggesting that anti-DP donor-specific antibodies increase the risk of graft failure (Ciurea et al. 2011; Crocchiolo et al. 2009; Fleischhauer et al. 2006; Petersdorf et al. 2001; Shaw et al. 2007; Spellman et al. 2010; Zino et al. 2004; Fleischhauer et al. 2012). Likewise, anti-DP antibodies appear to play an important role in kidney transplant outcomes (Singh et al. 2010; Thaunat et al. 2009). In addition, numerous studies suggest that *DPB1* is associated with predisposition to infectious disease such as hepatitis B (Howell and Visvanathan 2009; Kamatani et al. 2009), autoimmune disorders such as multiple sclerosis (Begovich et al. 1990; Odum et al. 1988) and juvenile idiopathic arthritis (Hollenbach et al. 2010), and leukemia (Taylor et al. 2002). The very strong evidence for a role of *DPB1* in development of chronic beryllium disease (CBD; Amicosante et al. 2001; Fontenot et al. 2000; Lombardi et al. 2001) suggests that despite low levels of cell surface expression, antigen presentation by the DP molecule is capable of stimulating a robust and clinically significant immune response.

Here, we present results for *DPA1* and *DPB1* four-digit allele-level typing in a large (*n* = 5,944) sample of unrelated European American stem cell donors previously characterized for other class I and class II loci (Maiers et al. 2007). Examination of genetic data for both chains of the DP heterodimer in the largest cohort to date, at the amino acid epitope, allele, genotype, and haplotype level, allows new insights into the functional units of selection and association for the DP heterodimer.

## Methods

### Subjects

The study cohort consisted of 5,944 (self-identified) European Caucasian individuals who donated hematopoietic stem cells for unrelated transplants facilitated by the National Marrow Donor Program (NMDP) during the years 1988 to 2003. Primary granulocyte and mononuclear cell preparations and transformed B cell lines from donors were distributed from the NMDP Research Sample Repository (Blood Systems Research Institute, San Francisco, CA) to nine laboratories for DNA preparation and HLA genotyping.

### Genotyping

Samples were genotyped at allele level for *DPA1* and *DPB1* (results from this same cohort for other loci: *HLA-A*, *HLA-B*, *HLA-C*, *HLA-DRB1*, and *HLA-DQB1* were reported previously; Klitz et al. 2003; Maiers et al. 2007). Sequence-specific oligonucleotide typing assays were performed as previously described (Williams et al. 2008) using reagents from the local laboratory, the 11th and 12th international workshops (Bignon and Fernandez-Vina 1997), and commercial vendors (Steiner et al. 2000). Sequence-based typing for *DPA1* was based on a 1,366-nucleotide PCR product including exons 2 through 4 as described previously (Rozemuller et al. 1995). DPB1 generic sequencing was based on a 574-nucleotide PCR product including exon 2 prepared from genomic DNA (Versluis et al. 1993) with heterozygous ambiguity resolved by allele-specific amplification or PCR-SSP.

The 5,944 samples were genotyped by nine different laboratories between 1994 and 2004. In order to maintain consistent reporting during the course of this project, results were interpreted to only consider *DPA1* and *DPB1* alleles identified in the 1994 HLA Nomenclature Report (Bodmer et al. 1994). One thousand six hundred seventy-one (28.1 %) individuals (3,342 chromosomes) were genotyped by two different laboratories for quality control; the remaining 4,273 (71.9 %) individuals were genotyped by a single laboratory.

### Resolution of discrepant results and ambiguous genotypes

The results were transferred from the nine laboratories via electronic files to the NMDP data center for interlaboratory comparison and also comparison with any typing previously reported by the transplant center (when available). Sixty-eight individuals (1.1 %) had previously reported results for *DPB1*, and two (0.03 %) had previously reported results for *DPA1*. A total of 20 out of 3,342 (0.6 %) discrepancies for *DPB1* and 9 out of 3,342 (0.26 %) for *DPA1* were identified and subsequently resolved. Etiology and resolution of discrepancies for this cohort generally followed the categories reported for a largely overlapping subset of *N* = 2,578 donors (Williams et al. 2008).

### Definition of serological and immunogenic epitopes of DPB1

Cano and Fernandez-Vina (2009) described two sets dimorphic amino acid epitopes at positions 56 and 85–87 that together accounted for the majority of DP serological reactivity observed in a sample of solid organ transplant patients. The first sequence dimorphism is found at DPB1 amino acid position 56, with either Ala (A) or Glu (E) at this site. The second variable region corresponds to amino acid positions 85–87 in the hypervariable region (HVR) “F,” and these amino acids are in complete LD; most *DPB1* alleles will have either the EAV (Glu-Ala-Val) or GPM (Gly-Pro-Met) motif in these positions. Combined, these two sequence dimorphisms were found to yield four serological specificities, defined as DP1 (56A; 85–87 EAV), DP2 (56E; 85–75 GPM), DP3 (56E; 85–87 EAV), and DP4 (56A; 85–87 GPM). In addition to standard analysis at the *DPB1* allele level, analysis was performed individually for these amino acid motifs, as well as for the four serological epitopes.

An alternative epitope, recognized by T cells, was defined in Zino et al. (2004, 2007) and Fleischhauer et al. (2012) on the basis of alloreactive T cell cross-reactivity patterns and is therefore referred to as T cell epitope (TCE). The TCE has so far not been mapped to defined structural amino acid residues, but was surmised to impact T cell alloreactivity via variable peptide presentation and shown to determine clinically nonpermissive mismatches for DPB1 in unrelated HSCT (Zino et al. 2004, 2007). The TCE is shared by defined subsets of DPB1 alleles, and allows alleles at this locus to be assigned to three categories of immunogenicity (highly immunogenic group 1 including DPB1*09:01, 10:01, 17:01; intermediately immunogenic group 2 including DPB1*03:01, 14:01, 45:01, 86:01, 104:01; and poorly immunogenic group 3 including most other alleles; Supplemental Table 2). In the present study, DPB1 alleles were categorized according to the TCE model outlined in Zino et al. (2004, 2007) and analyzed on that basis.

### Statistical analysis and data visualization

#### Hardy–Weinberg equilibrium

Fit of the data to Hardy–Weinberg expectations was assessed using both an exact test (Guo and Thompson 1992) and a standard goodness-of-fit (chi-squared) test implemented in the PyPop software package (Lancaster et al. 2003).

#### Homozygosity statistic

The homozygosity *F* statistics of Watterson (1978), calculated as the expected proportion of homozygotes under Hardy–Weinberg, was used as a measure of the allele and amino acid epitope frequency distributions and compared to the distributions expected under the neutral:

$$ F = \sum {p_i^2,} $$

where *p*_*i*_ is the frequency of the *i*th allele at a locus. The test is based on the observed number of alleles (*k*) at a locus and sample size (2*n*). The homozygosity test was applied using the exact test described by Slatkin (1994) and implemented in the PyPop software package (Lancaster et al. 2003).

#### Haplotype and disequilibrium estimation

Haplotype estimation was accomplished using an expectation–maximization algorithm which assigns population-level haplotype frequencies using simultaneous maximum likelihood estimation of *n* locus haplotype frequencies. Haplotypes were estimated for *DPA1* and *DPB1* alleles, as well as for *DPA1* alleles and *DPB1* amino acid and T cell epitopes.

A pairwise linkage disequilibrium statistic was calculated for each allele∼allele or allele∼epitope haplotype:

$$ {D_{{ij}}} = {x_{{ij}}} - {p_i}{q_j}{,} $$

where *x*_*ij*_ is the estimated haplotype frequency and *p*_*i*_ and *q*_*j*_ are the *i*th and *j*th allele frequencies at the two loci. To account for differing allele or epitope frequencies at the loci, a normalized disequilibrium value was used:

$$ D_{{ij}}^{\prime } = {D_{{ij}}}/{D_{{\max }}}, $$

where *D*_max_ is the lesser of *p*_*i*_ *q*_*j*_ and (1 − *p*_*i*_)(1 − *q*_*j*_), when *D*_*ij*_ is <0 and *p*_*i*_(1 − *q*_*j*_) and *q*_*j*_(1 − *p*_*i*_), when *D*_*ij*_ is >0.

A global disequilibrium statistic was also used to summarize disequilibrium at all possible haplotypes for two loci (Klitz et al. 1995):

$$ W = {\left( {\sum {\sum {D_{{ij}}^2/{p_i}{q_j}} } } \right)^{{1/2}}}, $$

where *p*_*i*_ and *q*_*j*_ are the observed allele frequencies at each of the two loci having *k* and *l* alleles, respectively. A normalized *W* was calculated to address differing numbers of alleles at the different loci:

$$ {W_n} = W/{\left( {\min \left( {k{,}l} \right) - 1} \right)^{{1/2}}}, $$

where *k* and *l* are the number of alleles at two loci (Cohen et al. 1988). The values of *W*_*n*_ fall between 0 and 1, and is identical to Cramer’s *V* (Cramer 1946).

Haplotype estimation was accomplished using the “haplo.em” function in the “haplo.stats” package (Sinnwell and Schaid 2009) for the R language for statistical computing (R Core Development Team 2009), version 2.9.2. All LD values were computed using the “ldkl” function in the “gap” package (Zhao and Tan 2009) for R. Clustered heatmaps of LD values were accomplished using the “heatmap” function in the base “stats” package for R, which utilizes a hierarchical similarity clustering procedure.

The DP heterodimer was visualized using Polyview 3D (Porollo and Meller 2007; http://polyview.cchmc.org/polyview3d.html) and input coordinates of the HLA-DP2 structure from Dai et al. (2010) in the Protein Data Bank (www.pdb.org; PDB ID code 3LQZ).

## Results

### *DPA1* and *DPB1* allele frequency distributions

Genotypic distributions for both *DPA1* and *DPB1* do not differ significantly from expectations under Hardy–Weinberg equilibrium. Allele frequencies for *DPA1* are given in Table 1. In this population, two of the nine *DPA1* alleles observed in the study cohort account for >95 % of the variation at this locus, with DPA1*01:03 *f* = 0.819 and DPA1*02:01 *f* = 0.140. Of the remaining alleles, only DPA1*02:02 is detected at a frequency greater than 1 %, and three of the nine alleles observed are found only once among the 11,888 chromosomes sampled here.

**Table 1:** *DPA1* allele frequencies in 5,944 individuals of European ancestry

*DPA1*	Position 31	Frequency
01:03	M	0.8186
01:04	M	0.0053
01:05	M	0.0001
02:01	Q	0.1403
02:02	Q	0.0343
02:03	M	0.0001
03:01	M	0.0011
03:02	M	0.0001
04:01	M	0.0001

The amino acid residue at position 31 is given for each allele

As expected, more diversity is observed for *DPB1* (Table 2), where 33 distinct *DPB1* alleles are seen in this cohort, but a single allele predominates and a handful of other alleles are present at moderate frequencies. Observations are consistent with those in numerous other European Caucasian populations, where DPB1*04:01 is the single most common allele (*f* = 0.439). Three remaining alleles (DPB1*02:01, DPB1*04:02, and DPB1*03:01) are seen at frequencies >10 %, while eight more alleles (DPB1*01:01, DPB1*11:01, DPB1*05:01, DPB1*06:01, DPB1*10:01, DPB1*13:01, DPB1*17:01, and DPB1*14:01, in order of decreasing frequency) have frequencies >1 %. Similar to the results for *DPA1*, a little less than one third (9 of 33) of the alleles are observed only as singleton copies.

**Table 2:** Frequencies of *DPB1* alleles

*DPB1*	Frequency	Position 56	Positions 85–87	Serological epitope	TCE group
04:01	0.4385	A	GPM	4	3
02:01	0.1274	E	GPM	2	3
04:02	0.1152	E	GPM	2	3
03:01	0.1011	E	EAV	3	2
01:01	0.0506	A	EAV	1	3
11:01	0.0229	A	EAV	1	3
05:01	0.0205	A	EAV	1	3
06:01	0.0183	E	EAV	3	3
10:01	0.0166	E	EAV	3	1
13:01	0.0157	A	EAV	1	3
17:01	0.0150	E	EAV	3	1
14:01	0.0117	E	EAV	3	2
19:01	0.0072	A	EAV	1	3
09:01	0.0072	E	EAV	3	1
15:01	0.0071	A	GPM	4	3
02:02	0.0067	A	GPM	4	3
20:01	0.0057	E	EAV	3	3
23:01	0.0054	A	GPM	4	3
16:01	0.0052	E	EAV	3	3
34:01	0.0006	A	GPM	4	3
35:01	0.0003	E	EAV	3	NC
45:01	0.0003	E	EAV	3	2
33:01	0.0002	A	GPM	4	3
39:01	0.0002	A	GPM	4	3
26:01	0.0001	A	EAV	1	NC
30:01	0.0001	A	EAV	1	NC
36:01	0.0001	A	EAV	1	NC
54:01	0.0001	A	EAV	1	NC
87:01	0.0001	A	EAV	1	NC
18:01	0.0001	E	GPM	2	NC
51:01	0.0001	E	GPM	2	3
72:01	0.0001	A	GPM	4	3
99:01	0.0001	A	GPM	4	3

Amino acid motifs for positions 56 and 85–87 are given for each allele. The serological epitope DP1–DP4 described in Cano and Fernandez-Vina (2009) is given along with the T cell receptor group defined in Zino et al. (2004)

*NC* not classified

### *DPB1* serological epitopes

The frequencies in the study population of the amino acid epitopes at positions 56 and positions 85–87 are shown in Table 3. Position 56 is marked by extremely balanced frequencies of 0.576 for alanine at this position and 0.424 for glutamic acid. The amino acids within the second motif examined here, at positions 85–87 in the HVR “F,” are in complete LD, with one of two epitopes in all *DPB1* alleles in this sample: either EAV (*f* = 0.30) or GPM (*f* = 0.70). Together, these two sequence dimorphisms correspond to four serological specificities: DP1, DP2, DP3, and DP4; the frequencies of these are given in Table 4.

**Table 3:** Frequency of polymorphic amino acids at *DPB1* positions 56 and 85–87

DPB1 amino acid residue	Frequency
85–87 EAV	0.300
85–87 GPM	0.700
56A	0.576
56E	0.424

**Table 4:** Amino acid motifs and frequencies for serological epitopes DP1–DP4

Serological epitope	Position 56 motif	Positions 85–87 motif	Frequency
DP1	A	EAV	0.117
DP2	E	GPM	0.243
DP3	E	EAV	0.181
DP4	A	GPM	0.459

### *DPB1* TCE

When the data for this cohort are examined with respect to the TCE immunogenicity groups defined by Zino et al. (2004, 2007), it is apparent that the immunogenic TCE group 1 and 2 alleles together account for only 15.2 % of observed alleles (Table 5). TCE group 1 alleles all bear the “EAV” motif in positions 85–87, and are all in LD with DPA1*02:01. However, neither the 85–87 EAV nor the presence of DPA1*02:01 was specific for alleles from TCE group 1 as both are found also in some alleles from TCE groups 2 and 3 (Table 5 and Supplemental Table 2).

**Table 5:** Frequency of TCE groups (Zino et al. 2004) and amino acid positions 85–87 motifs within each group

TCE group	Frequency	Frequency position 85–87 EAV within group
1	0.0387	1
2	0.1131	0.8
3	0.8474	0.328
Not classified	0.0008	0.889

### Homozygosity statistic

Calculation of Watterson’s homozygosity statistic (*F*) for both *DPA1* and *DPB1* reveals that the allele frequency distributions for both loci do not differ significantly from expectations under a neutral model (Table 6). In contrast, when the frequency distributions for *DPB1* are analyzed with respect to the four serological specificities, DP1–DP4 (Cano and Fernandez-Vina 2009) are significantly more even than expected under neutrality (*p* < 0.005), suggesting evidence for balancing selection for these specificities (Table 6). The frequency distributions of DPB1 alleles in the three TCE groups, on the other hand, do not depart significantly from neutral expectations.

**Table 6:** Homozygosity statistic (*F*) for DPA1, DPB1, and DPB1 defined in terms of serological epitopes (Cano and Fernandez-Vina 2009) or T cell epitopes (TCE; Zino et al. 2004)

Locus/epitope group	*F* (obs)	*F* (exp)	*p*
DPA1	0.691	0.517	0.798
DPB1	0.237	0.195	0.784
DPB1 serological groups P1–P4	0.316	0.748	0.0035
DPB1 TCE groups 1–3	0.734	0.817	0.318

### *DPA1*∼*DPB1* haplotypic associations and linkage disequilibrium

Population-level estimates for all possible *DPA1-DPB1* haplotypes are given in Supplemental Table 1. Three haplotypes, DPA1*01:03∼DPB1*02:01 (*f* = 0.124), DPA1*01:03∼DPB1*04:01 (*f* = 0.433), and DPA1*01:03∼DPB1*04:02 (*f* = 0.114) account for more than two thirds of the observed haplotypes in this cohort. Table 7 shows the 15 most frequent haplotypes with a cumulative frequency of >95 %. The normalized LD (*D*′) statistics for these haplotypes show extensive LD between *DPA1* and *DPB1* alleles; the notable exception is DPA1*02:02∼DPB1*01:01, which is observed in this cohort at a frequency of less than 1 %. The overall (global) linkage disequilibrium (*W*_*n*_) = 0.515 for *DPA1∼DPB1* associations.

**Table 7:** Frequency and linkage disequilibrium of the 15 most common *DPA1*∼*DPB1* haplotypes

Haplotype	*DPA1* position 31	*DPB1* position 85–87	Count	Frequency	*D*′
DPA1*01:03~DPB1*04:01	M	GPM	5,146.34	0.4329	0.9295
DPA1*01:03~DPB1*02:01	M	GPM	1,470.19	0.12367	0.8404
DPA1*01:03~DPB1*04:02	M	GPM	1,350.52	0.1136	0.9216
DPA1*01:03~DPB1*03:01	M	EAV	1,189.94	0.1001	0.9447
DPA1*02:01~DPB1*01:01	Q	EAV	506.21	0.04258	0.8165
DPA1*02:01~DPB1*11:01	Q	EAV	268.98	0.02263	0.9871
DPA1*01:03~DPB1*06:01	M	EAV	216	0.01817	0.9746
DPA1*02:01~DPB1*10:01	Q	EAV	191.97	0.01615	0.9703
DPA1*02:02~DPB1*05:01	Q	EAV	180.66	0.0152	0.7312
DPA1*02:01~DPB1*17:01	Q	EAV	173.97	0.01463	0.9737
DPA1*02:01~DPB1*13:01	Q	EAV	162.04	0.01363	0.8502
DPA1*02:01~DPB1*14:01	Q	EAV	137.99	0.01161	0.9916
DPA1*02:02~DPB1*01:01	Q	EAV	86.49	0.00728	0.17
DPA1*02:01~DPB1*09:01	Q	EAV	85	0.00715	1
DPA1*02:02~DPB1*19:01	Q	EAV	82.99	0.00698	0.9755

The amino acid motifs at DPA1 position 31 and DPB1 positions 85–87 are given

**Fig. 1:**
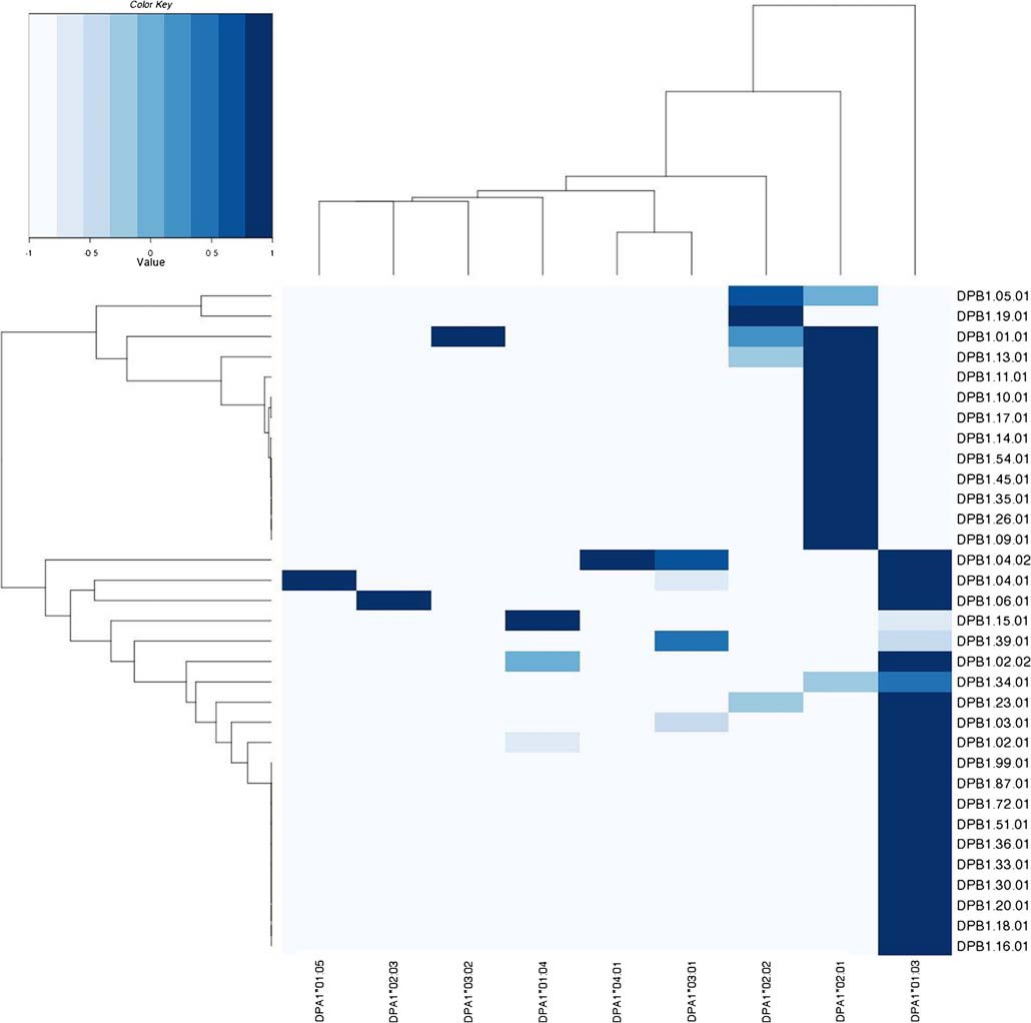
A clustered heatmap for *DPA1*∼*DPB1* haplotype normalized linkage disequilibrium values (*D*′). *DPA1* alleles are shown along the *x*-axis, and *DPB1* alleles are shown on the *y*-axis

When *DPA1-DPB1* haplotypes are examined with respect to *DPB1* amino acid motifs, rather than alleles, the patterns of association suggest that the *DPA1* allele that is associated with *DPB1* is strictly related to *DPB1* polymorphisms at amino acid positions 85–87 in the HVR “F.” Near complete LD (*D*′ = 0.94, *p* < 0.05) is observed between DPA1*0201 and *DPB1* alleles with the EAV motif, while DPA1*0103 nearly always occurs on a haplotype with the DPB1 85–87 GPM motif (*D*′ = 0.87, *p* < 0.01). The global LD value (*W*_*n*_) between *DPA1* and positions 85–87 = 0.65. These relationships are evident in Fig. 1, which shows a clustered heatmap for *DPA1∼DPB1* haplotype *D*′ values. *DPB1* alleles break down into two primary clusters, which are nearly strictly defined by polymorphisms at positions 85–87.

These results for LD between *DPA1* alleles and *DPB1* amino acid motifs stand in stark contrast to the patterns of LD observed between amino acid motifs within *DPB1*. Here, we find that very little LD exists between the pair of sequence dimorphisms at positions 56 and 85–87, corresponding to the serological specificities described above, with *W*_*n*_ = 0.242.

## Discussion

The high levels of linkage disequilibrium between *DPA1* and *DPB1* suggest the possibility of nonpermissive combinations for the heterodimer, similar to that suggested for the DQ molecule. It has been shown that for the heterodimer encoded by *DQA1-DQB1*, certain alpha–beta combinations are unstable at the cell surface, and these have been associated with the patterns of LD for these genes. The patterns of LD observed here are consistent with the notion that particular combinations of DP alpha and beta chains may be structurally impermissible. In the DP heterodimer, positions 85–87 are thought to be important primarily in interaction with the alpha chain, as well as participating in the P1 pocket (Diaz et al. 2003). This is analogous to the more well-characterized *DRB1* protein structure: DPB1 position 84, in LD with positions 85–87, corresponds to *DRB1* position 86, which is thought to contribute to both dimer stability (Verreck et al. 1993) and the position of bound peptide in the P1 pocket, impacting the MHC–peptide conformation (Wu and Gorski 1997).

**Fig. 2:**
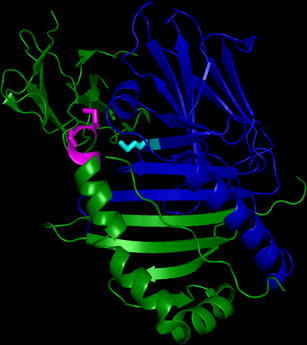
The crystal structure for the DP2 molecule, with position 31 on *DPA1* and positions 84–87 for *DPB1*, and their side chains, highlighted (position 31 (DPA1)—*cyan*; positions 84–87 (DPB1)—*magenta*)

While significantly less polymorphic than *DRB1*, the patterns of LD and amino acid variation for the *DPB1* locus lend further support to the importance of the P1 pocket in driving *DPA1∼DPB1* LD. In contrast to DPB1, DPA1 polymorphism is extremely limited and restricted to only a handful of amino acid sites. Examination of the amino acid sequences for the DPA1 alleles reveals that a single amino acid polymorphism at position 31 [methionine (M) or glutamine (Q)] subdivides the alleles at this locus along the lines of the patterns of LD (Table 1). Position 31, like positions 85–87 in *DPB1*, participates in the P1 pocket. Figure 2 shows the crystal structure for DP2 (Dai et al. 2010) with position 31 on *DPA1* and positions 84–87 for *DPB1*, and their side chains, highlighted. The structure makes clear the critical role of these residues in the P1 pocket of the peptide-binding region, as well as interaction between the alpha and beta chains. While beta chain positions 84–87, located within the peptide-binding region alpha helix, is antigenic and most likely is in contact with bound peptide and the T cell receptor, position 31 on the alpha chain, forms part of the beta-pleated sheet that forms the floor of the peptide-binding groove and is not exposed to the TCR or solvent.

The critical role of position 31 in determining LD with *DPB1* alleles is illustrated by the rare DPA1*01:06:02 allele, first characterized in an individual from Kenya (Peterson et al. 2008). The novel allele was initially detected due to the observation of heterozygosity at position 31 (methionine→glutamine) for a genotyping otherwise consistent with DPA1*01:03 homozygous. Glutamine at position 31 is one of two amino acid positions delineating the DPA1*02:01 and 02:02 alleles, which are in near complete LD with *DPB1* alleles bearing the position 85–87 “EAV” motif. Interestingly, the Kenyan individual in whom this allele was identified was heterozygous at *DPB1*, with the “EAV” motif present in one allele, DPB1*01:01:01, but not the other, DPB1*02:01:02.

While the allele frequency distributions for *DPA1* and *DPB1* do not differ significantly from neutral expectations, the frequencies for the broad serological types DP1–DP4 are significantly more even than expected under neutrality, suggesting evidence for balancing selection for these specificities. These findings are in keeping with those from other studies in multiple human populations, where, unlike other class II loci, the *DPB1* locus did not show evidence for balancing selection at the allele level. In most populations studied to date, *DPB1* frequency distributions did not differ significantly from expectations under neutrality (Begovich et al. 2001; Salamon et al. 1999; Solberg et al. 2008), or showed evidence of directional, or purifying selection (Hollenbach et al. 2001; Pérez-Miranda et al. 2004). However, when the data in Salamon et al. (1999) were examined at the amino acid level, several amino acid sites were found to have significantly balanced polymorphism; notably, the most balanced sites were found to be positions 55–56 and 84–87, consistent with the findings in the present study. Salamon et al. concluded that selection may be operating at the amino acid level in *DPB1*, and that because this locus is characterized by polymorphism primarily related to gene conversion events, resulting in lower overall polymorphism, this selective effect is masked at the allele level. More recent work by Mack (2011, personal communication) has confirmed that positions 55–56 and 84–87 appear to be particularly balanced for *DPB1* in most populations worldwide, regardless of whether the *DPB1* allele frequencies are even or directionally skewed in the population.

Strikingly, there is more evidence of recombination between *DPB1* positions 56 and 85–87 (*W*_*n*_ = 0.28), within the gene, than between *DPA1* and *DPB1* positions 85–87 (*W*_*n*_ = 0.65), i.e., between two adjacent genes. The finding of minimal LD between *DPB1* positions 56 and 85–87 is in keeping with numerous studies describing evidence for a history of extensive recombination within the *DPB1* locus. While gene conversion and recombination are thought to be an important factor in HLA polymorphism, Buhler and Sanchez-Mazas (2011) have noted that HLA-*DPB1* appears to have been particularly impacted by gene conversion relative to other HLA loci, and *DPB1* alleles are much more closely related to each other than alleles of other loci; the authors concluded that the patterns of allele and amino acid frequency distributions in world populations show evidence of ancient, rather than recent, balancing selection.

It is interesting to note that the position 56 and 85–87 motifs appear to be characteristics of a specific DP supertype defined by an unusually similar peptide-binding motif (Sidney et al. 2010) and identified among most common *DPB1* alleles in most human populations. While the supertype largely shares specificity in the main P6 pocket of the peptide-binding region, the position 85–87 motif impacts P1 specificity. An alternative peptide-binding motif for DPB1*09:01 (Dong et al. 1995) is markedly different from that for the common DP supertype that includes DPB1*02:01 and DPB1*04:01. Interestingly, DPB1*09:01 is the prototype allele of the highly immunogenic TCE group 1 in the original paper by Zino et al. (2004). The TCE group 1 alleles all possess the EAV motif at positions 85–87, and in this study were always observed with DPA1*02:01, consistent with observations for all *DPB1* alleles with this motif. It is tempting to speculate that the strong alloreactivity to this TCE group demonstrated both clinically and in mixed lymphocyte culture reactions in vitro (Sizzano et al. 2010; Crocchiolo et al. 2009; Zino et al. 2004; Fleischhauer et al. 2012) could be correlated with the presence of this motif in association with the *DPA1* linkage. Likewise, the very strong association of *DPB1* with CBD has been pinpointed to a specific role for glutamic acid at amino acid position 69, suggesting that for *DPB1* the amino acid residue, rather than allelic identity, may be the important unit of association in human disease.

Taken together, the data in this study suggest that for the *DPA1-DPB1* heterodimer, the unit of selection is the combined amino acid epitope contributed by both the *DPA1* and *DPB1* genes, rather than the allele, and that patterns of LD are driven primarily by dimer stability and conformation of the P1 pocket. This may help explain the differential pattern of allele frequency distribution observed for this locus relative to the other class II loci. These findings further support the notion that allele-level associations in disease and transplantation may not be the most important unit of analysis, and that they should be considered instead in the molecular context.

## Electronic supplementary materials

Below is the link to the electronic supplementary material.

ESM 1 (DOC 73 kb)

ESM 2 (DOC 84 kb)

## References

[CR1] Amicosante M, Sanarico N, Berretta F, Arroyo J, Lombardi G, Lechler R, Colizzi V, Saltini C (2001). Beryllium binding to HLA-DP molecule carrying the marker of susceptibility to berylliosis glutamate beta 69. Hum Immunol.

[CR2] Begovich AB, Helmuth RC, Oksenberg JR, Sakai K, Tabira T, Sasazuki T, Steinman L, Erlich HA (1990). HLA-DP beta and susceptibility to multiple sclerosis: an analysis of caucasoid and Japanese patient populations. Hum Immunol.

[CR3] Begovich AB, McClure GR, Suraj VC, Helmuth RC, Fildes N, Bugawan TL, Erlich HA, Klitz W (1992). Polymorphism, recombination and linkage disequilibrium within the HLA class II region. J Immunol.

[CR4] Begovich AB, Moonsamy PV, Mack SJ, Barcellos LF, Steiner LL, Grams S, Suraj-Baker V, Hollenbach J, Trachtenberg E, Louie L, Zimmerman P, Hill AV, Stoneking M, Sasazuki T, Konenkov VI, Sartakova ML, Titanji VP, Rickards O, Klitz W (2001). Genetic variability and linkage disequilibrium within the HLA-DP region: analysis of 15 different populations. Tissue Antigens.

[CR5] Bignon JD and Fernandez-Vina M (1997) Protocols for the 12th International Histocompatibility Workshop for typing of HLA class II alleles by DNA amplification by the polymerase chain reaction (PCR) and hybridization with sequence specific oligonucleotide probes (SSOP). In Charron D (ed): Proceedings of the 12th International Histocompatibility Workshop and Conference, pp. 1–777, EDK, Paris

[CR6] Bodmer JG, Marsh SG, Albert ED, Bodmer WF, Dupont B, Erlich HA, Mach B, Mayr WR, Parham P, Sasazuki T (1994). Nomenclature for factors of the HLA system, 1994. Tissue Antigens.

[CR7] Bugawan TL, Klitz W, Blair A, Erlich HA (2000). High-resolution HLA class I typing in the CEPH families: analysis of linkage disequilibrium among HLA loci. Tissue Antigens.

[CR8] Buhler S, Sanchez-Mazas A (2011). HLA DNA sequence variation among human populations: molecular signatures of demographic and selective events. PLoS One.

[CR9] Cano P, Fernandez-Vina M (2009). Two sequence dimorphisms of DPB1 define the immunodominant serologic epitopes of HLA-DP. Hum Immunol.

[CR10] Ciurea SO, Thall PF, Wang X, Wang SA, Hu Y, Cano P, Aung F, Rondon G, Molldrem JJ, Korbling M, Shpall EJ, de Lima M, Champlin RE, Fernandez-Vina M (2011). Donor-specific anti-HLA antibodies and graft failure in matched unrelated donor hematopoietic stem cell transplantation. Blood.

[CR11] Cohen J (1988). Statistical power analysis for the behavioral sciences.

[CR12] Cramer H (1946). Mathematical methods of statistics.

[CR13] Crocchiolo R, Zino E, Vago L, Oneto R, Bruno B, Pollichieni S, Sacchi N, Sormani MP, Marcon J, Lamparelli T, Fanin R, Garbarino L, Miotti V, Bandini G, Bosi A, Ciceri F, Bacigalupo A, Fleischhauer K (2009). Nonpermissive HLA-DPB1 disparity is a significant independent risk factor for mortality after unrelated hematopoietic stem cell transplantation. Blood.

[CR14] Dai S, Murphy GA, Crawford F, Mack DG, Falta MT, Marrack P, Kappler JW, Fontenot AP (2010). Crystal structure of HLA-DP2 and implications for chronic beryllium disease. Proc Natl Acad Sci U S A.

[CR15] Diaz G, Amicosante M, Jaraquemada D, Butler RH, Guillen MV, Sanchez M, Nombela C, Arroyo J (2003). Functional analysis of HLA-DP polymorphism: a crucial role for DPbeta residues 9, 11, 35, 55, 56, 69 and 84–87 in T cell allorecognition and peptide binding. Int Immunol.

[CR16] Sinnwell JP and Schaid DJ (2009) haplo.stats: statistical analysis of haplotypes with traits and covariates when linkage phase is ambiguous. http://mayoresearch.mayo.edu/mayo/research/schaid_lab/software.cfm

[CR17] Dong RP, Kamikawaji N, Toida N, Fujita Y, Kimura A, Sasazuki T (1995). Characterization of T cell epitopes restricted by HLA-DP9 in streptococcal M12 protein. J Immunol.

[CR18] Edwards JA, Durant BM, Jones DB, Evans PR, Smith JL (1986). Differential expression of HLA class II antigens in fetal human spleen: relationship of HLA-DP, DQ, and DR to immunoglobulin expression. J Immunol.

[CR19] Fernandez-Vina MA, Gao XJ, Moraes ME, Moraes JR, Salatiel I, Miller S, Tsai J, Sun YP, An JB, Layrisse Z (1991). Alleles at four HLA class II loci determined by oligonucleotide hybridization and their associations in five ethnic groups. Immunogenetics.

[CR20] Fleischhauer K, Locatelli F, Zecca M, Orofino MG, Giardini C, De Stefano P, Pession A, Iannone AM, Carcassi C, Zino E, La Nasa G (2006). Graft rejection after unrelated donor hematopoietic stem cell transplantation for thalassemia is associated with nonpermissive HLA-DPB1 disparity in host-versus-graft direction. Blood.

[CR21] Fleischhauer K, Shaw BE, Gooley T, Malkki M, Bardy P, Bignon JD, Dubois V, Horowitz MM, Madrigal JA, Morishima Y, Oudshoorn M, Ringden O, Spellman S, Velardi A, Zino E, Petersdorf EW (2012) Effect of T-cell-epitope matching at HLA-DPB1 in recipients of unrelated donor haemopoietic-cell transplantation: a retrospective study. Lancet Oncol. doi:10.1016/s1470-2045(12)70004-9 10.1016/S1470-2045(12)70004-9 PMC3813000 22340965

[CR22] Fontenot AP, Torres M, Marshall WH, Newman LS, Kotzin BL (2000). Beryllium presentation to CD4+ T cells underlies disease-susceptibility HLA-DP alleles in chronic beryllium disease. Proc Natl Acad Sci U S A.

[CR23] Gallardo D, Brunet S, Torres A, Alonso-Nieto M, Vallejo C, Jimenez A, Gonzalez M, Sanz G, Serrano D, Espigado I, Osorio S, Carreras E, Martiin C, Sanz-Rodriguez C, Sierra J, Zuazu J, Gonzalez-Escribano MF, Gonzalez JR, Roman J, De Oteyza JP, De La Camara R (2004). Hla-DPB1 mismatch in HLA-A-B-DRB1 identical sibling donor stem cell transplantation and acute graft-versus-host disease. Transplantation.

[CR24] Gendzekhadze K, Herrera F, Montagnani S, Balbas O, Witter K, Albert E, Layrisse Z (2004). HLA-DP polymorphism in Venezuelan Amerindians. Hum Immunol.

[CR25] Guardiola J, Maffei A (1993). Control of MHC class II gene expression in autoimmune, infectious, and neoplastic diseases. Crit Rev Immunol.

[CR26] Guo SW, Thompson EA (1992). Performing the exact test of Hardy–Weinberg proportion for multiple alleles. Biometrics.

[CR27] Hollenbach JA, Thompson SD, Bugawan TL, Ryan M, Sudman M, Marion M, Langefeld CD, Thomson G, Erlich HA, Glass DN (2010). Juvenile idiopathic arthritis and HLA class I and class II interactions and age-at-onset effects. Arthritis Rheum.

[CR28] Hollenbach JA, Thomson G, Cao K, Fernandez-Vina M, Erlich HA, Bugawan TL, Winkler C, Winter M, Klitz W (2001). HLA diversity, differentiation, and haplotype evolution in Mesoamerican Natives. Hum Immunol.

[CR29] Howell JA, Visvanathan K (2009). A novel role for human leukocyte antigen-DP in chronic hepatitis B infection: a genomewide association study. Hepatology.

[CR30] Kamatani Y, Wattanapokayakit S, Ochi H, Kawaguchi T, Takahashi A, Hosono N, Kubo M, Tsunoda T, Kamatani N, Kumada H, Puseenam A, Sura T, Daigo Y, Chayama K, Chantratita W, Nakamura Y, Matsuda K (2009). A genome-wide association study identifies variants in the HLA-DP locus associated with chronic hepatitis B in Asians. Nat Genet.

[CR31] Klitz W, Maiers M, Spellman S, Baxter-Lowe LA, Schmeckpeper B, Williams TM, Fernandez-Vina M (2003). New HLA haplotype frequency reference standards: high-resolution and large sample typing of HLA DR-DQ haplotypes in a sample of European Americans. Tissue Antigens.

[CR32] Klitz W, Stephen JC, Grote M, Carrington M (1995). Discordant patterns of linkage disequilibrium of the peptide transporter loci within the HLA class II region. Am J Hum Genet.

[CR33] Kwok WW, Kovats S, Thurtle P, Nepom GT (1993). HLA-DQ allelic polymorphisms constrain patterns of class II heterodimer formation. J Immunol.

[CR34] Kwok WW, Thurtle P, Nepom GT (1989). A genetically controlled pairing anomaly between HLA-DQ alpha and HLA-DQ beta chains. J Immunol.

[CR35] Lancaster A, Nelson MP, Meyer D, Thomson G (2003) PyPop: a software framework for population genomics: analyzing large-scale multi-locus genotype data. Pac Symp Biocomput 514–525 PMC3891851 12603054

[CR36] Lombardi G, Germain C, Uren J, Fiorillo MT, du Bois RM, Jones-Williams W, Saltini C, Sorrentino R, Lechler R (2001). HLA-DP allele-specific T cell responses to beryllium account for DP-associated susceptibility to chronic beryllium disease. J Immunol.

[CR37] Maiers M, Gragert L, Klitz W (2007). High-resolution HLA alleles and haplotypes in the United States population. Hum Immunol.

[CR38] Odum N, Hyldig-Nielsen JJ, Morling N, Sandberg-Wollheim M, Platz P, Svejgaard A (1988). HLA-DP antigens are involved in the susceptibility to multiple sclerosis. Tissue Antigens.

[CR39] Pérez-Miranda AM, Alfonso-Sánchez M, Vidales MC, Calderón R, Peña JA (2004). Genetic polymorphism and linkage disequilibrium of the HLA-DP region in Basques from Navarre (Spain). Tissue Antigens.

[CR40] Petersdorf EW, Gooley T, Malkki M, Anasetti C, Martin P, Woolfrey A, Smith A, Mickelson E, Hansen JA (2001). The biological significance of HLA-DP gene variation in haematopoietic cell transplantation. Br J Haematol.

[CR41] Peterson TA, Luo M, Mao X, Brunham RC, Plummer FA (2008). Identification of a novel DPA1 allele, DPA1*010602, in an East African population. Hum Immunol.

[CR42] Porollo A, Meller J (2007). Versatile annotation and publication quality visualization of protein complexes using POLYVIEW-3D. BMC Bioinformatics.

[CR43] Rozemuller EH, Bouwens AG, van Oort E, Versluis LF, Marsh SG, Bodmer JG, Tilanus MG (1995). Sequencing-based typing reveals new insight in HLA-DPA1 polymorphism. Tissue Antigens.

[CR44] Salamon H, Klitz W, Easteal S, Gao X, Erlich HA, Fernandez-Vina M, Trachtenberg EA (1999). Evolution of HLA class II molecules: allelic and amino acid site variability across populations. Genetics.

[CR45] Shaw BE, Gooley TA, Malkki M, Madrigal JA, Begovich AB, Horowitz MM, Gratwohl A, Ringden O, Marsh SG, Petersdorf EW (2007). The importance of HLA-DPB1 in unrelated donor hematopoietic cell transplantation. Blood.

[CR46] Shaw BE, Marsh SG, Mayor NP, Russell NH, Madrigal JA (2006). HLA-DPB1 matching status has significant implications for recipients of unrelated donor stem cell transplants. Blood.

[CR47] Shaw BE, Potter MN, Mayor NP, Pay AL, Smith C, Goldman JM, Prentice HG, Marsh SG, Madrigal JA (2003). The degree of matching at HLA-DPB1 predicts for acute graft-versus-host disease and disease relapse following haematopoietic stem cell transplantation. Bone Marrow Transplant.

[CR48] Sidney J, Steen A, Moore C, Ngo S, Chung J, Peters B, Sette A (2010). Five HLA-DP molecules frequently expressed in the worldwide human population share a common HLA supertypic binding specificity. J Immunol.

[CR49] Singh P, Colombe BW, Francos GC, Martinez Cantarin MP, Frank AM (2010). Acute humoral rejection in a zero mismatch deceased donor renal transplant due to an antibody to an HLA-DP alpha. Transplantation.

[CR50] Sizzano F, Zito L, Crivello P, Crocchiolo R, Vago L, Zino E, Fleischhauer K (2010). Significantly higher frequencies of alloreactive CD4^+^ T cells responding to nonpermissive than to permissive HLA-DPB1 T-cell epitope disparities [letter]. Blood.

[CR51] Slatkin M (1994). An exact test for neutrality based on the Ewens sampling distribution. Genet Res.

[CR52] Slatkin M (2000). Balancing selection at closely linked, overdominant loci in a finite population. Genetics.

[CR53] Solberg OD, Mack SJ, Lancaster AK, Single RM, Tsai Y, Sanchez-Mazas A, Thomson G (2008). Balancing selection and heterogeneity across the classical human leukocyte antigen loci: a meta-analytic review of 497 population studies. Hum Immunol.

[CR54] Spellman S, Bray R, Rosen-Bronson S, Haagenson M, Klein J, Flesch S, Vierra-Green C, Anasetti C (2010). The detection of donor-directed, HLA-specific alloantibodies in recipients of unrelated hematopoietic cell transplantation is predictive of graft failure. Blood.

[CR55] Steiner L, Moonsamy PV, Bugawan TL, Begovich AB (2000) HLA-DPA1 and -DPB1 typing using the polymerase chain reaction and non-radioactive sequence-specific oligonucleotide probes. In Hahn A (ed.) ASHI laboratory manual. American Society for Histocompatibility and Immunogenetics: Mt. Laurel, pp. V.C.3.1–16

[CR56] Taylor GM, Dearden S, Ravetto P, Ayres M, Watson P, Hussain A, Greaves M, Alexander F, Eden OB (2002). Genetic susceptibility to childhood common acute lymphoblastic leukaemia is associated with polymorphic peptide-binding pocket profiles in *HLA-DPB1*0201*. Hum Mol Genet.

[CR57] R Core Development Team (2009). R: a language and environment for statistical computing.

[CR58] Thaunat O, Hanf W, Dubois V, McGregor B, Perrat G, Chauvet C, Touraine JL, Morelon E (2009). Chronic humoral rejection mediated by anti-HLA-DP alloantibodies: insights into the role of epitope sharing in donor-specific and non-donor specific alloantibodies generation. Transpl Immunol.

[CR59] Verreck FA, Termijtelen A, Koning F (1993). HLA-DRbeta chain residue 86 controls DRalpha-beta dimer stability. Eur J Immunol.

[CR60] Versluis LF, Rozemuller E, Tonks S, Marsh SG, Bouwens AG, Bodmer JG, Tilanus MG (1993). High-resolution HLA-DPB typing based upon computerized analysis of data obtained by fluorescent sequencing of the amplified polymorphic exon 2. Hum Immunol.

[CR61] Vrana M, Dobrovolna M, Cetkovsky P, Nazarova S, Vondrackova H, Sedlacek P (2006). HLA-DPB1 gene analysis in haematopoietic stem cell transplantations. Cas Lek Cesk.

[CR62] Watterson G (1978). The homozygosity test of neutrality. Genetics.

[CR63] Williams TM, Winden T, Setterholm M, Vierra-Green CA, Spellman S, Flesch S, Awdeh Z, Baxter-Lowe LA, Begovich AB, Fernandez-Vina M, Hegland J, Hurley CK, Johnson D, Noreen H, Salazar M, Schmeckpeper B, Yunis EJ (2008). Strategies and technical challenges in allele level class II typing in 2578 bone marrow transplantation donor-recipient pairs. Hum Immunol.

[CR64] Wu S, Gorski J (1997). Polymorphism at beta 85 and not beta 86 of HLA-DR1 is predominantly responsible for restricting the nature of the anchor side chain: implication for concerted effects of class II MHC polymorphism. Int Immunol.

[CR65] Zhao JH and Tan Q (2009) Integrated analysis of genetic data with R. Hum Genomics 2(4):258 10.1186/1479-7364-2-4-258 PMC3525150 16460651

[CR66] Zino E, Frumento G, Marktel S, Sormani MP, Ficara F, Di Terlizzi S, Parodi AM, Sergeant R, Martinetti M, Bontadini A, Bonifazi F, Lisini D, Mazzi B, Rossini S, Servida P, Ciceri F, Bonini C, Lanino E, Bandini G, Locatelli F, Apperley J, Bacigalupo A, Ferrara GB, Bordignon C, Fleischhauer K (2004). A T-cell epitope encoded by a subset of HLA-DPB1 alleles determines nonpermissive mismatches for hematologic stem cell transplantation. Blood.

[CR67] Zino E, Vago L, Di Terlizzi S, Mazzi B, Zito L, Sironi E, Rossini S, Bonini C, Ciceri F, Roncarolo MG, Bordignon C, Fleischhauer K (2007). Frequency and targeted detection of HLA-DPB1 T cell epitope disparities relevant in unrelated hematopoietic stem cell transplantation. Biol Blood Marrow Transplant.

