# Supplementary material for: A combined DPA1∼DPB1 amino acid epitope is the primary unit of selection on the HLA-DP heterodimer
Source: Immunogenetics. 2012 Apr 13;64(8):559–69. doi: 10.1007/s00251-012-0615-3 (PMC3395342; doi:10.1007/s00251-012-0615-3)
Supplement: Supplementary file 1 — (DOC 73 kb) [file 251_2012_615_MOESM1_ESM.doc]

Supplemental Table 1. Matrix of HLA-DPA1~DPB1 haplotype frequencies in a European American cohort.

|  | DPB1*01:01 | DPB1*02:01 | DPB1*02:02 | DPB1*03:01 | DPB1*04:01 | DPB1*04:02 | DPB1*05:01 | DPB1*06:01 | DPB1*09:01 | DPB1*10:01 | DPB1*11:01 |
| --- | --- | --- | --- | --- | --- | --- | --- | --- | --- | --- | --- |
| DPA1*01:03 | 0.00061 | 0.12367 | 0.00656 | 0.1001 | 0.4329 | 0.1136 | 0.00068 | 0.01817 | 0 | 0.00042 | 0.00017 |
| DPA1*01:04 | 0 | 0.00017 | 0.00008 | 0 | 0 | 0 | 0 | 0 | 0 | 0 | 0 |
| DPA1*01:05 | 0 | 0 | 0 | 0 | 0.00008 | 0 | 0 | 0 | 0 | 0 | 0 |
| DPA1*02:01 | 0.04258 | 0.00276 | 0.00008 | 0.00091 | 0.00256 | 0.00014 | 0.00465 | 0 | 0.00715 | 0.01615 | 0.02263 |
| DPA1*02:02 | 0.00728 | 0.00076 | 0 | 0.00004 | 0.00283 | 0.0006 | 0.0152 | 0 | 0 | 0 | 0.00008 |
| DPA1*02:03 | 0 | 0 | 0 | 0 | 0 | 0 | 0 | 0.00008 | 0 | 0 | 0 |
| DPA1*03:01 | 0 | 0 | 0 | 0.00006 | 0.00013 | 0.00081 | 0 | 0 | 0 | 0 | 0 |
| DPA1*03:02 | 0.00008 | 0 | 0 | 0 | 0 | 0 | 0 | 0 | 0 | 0 | 0 |
| DPA1*04:01 | 0 | 0 | 0 | 0 | 0 | 0.00008 | 0 | 0 | 0 | 0 | 0 |
| (cont) | DPB1*13:01 | DPB1*14:01 | DPB1*15:01 | DPB1*16:01 | DPB1*17:01 | DPB1*18:01 | DPB1*19:01 | DPB1*20:01 | DPB1*23:01 | DPB1*26:01 | DPB1*30:01 |
| DPA1*01:03 | 0.0016 | 0.00008 | 0.00202 | 0.00522 | 0.00034 | 0.00008 | 0.00017 | 0.00572 | 0.00525 | 0 | 0.00008 |
| DPA1*01:04 | 0 | 0 | 0.00505 | 0 | 0 | 0 | 0 | 0 | 0 | 0 | 0 |
| DPA1*01:05 | 0 | 0 | 0 | 0 | 0 | 0 | 0 | 0 | 0 | 0 | 0 |
| DPA1*02:01 | 0.01363 | 0.01161 | 0 | 0 | 0.01463 | 0 | 0 | 0 | 0 | 0.00008 | 0 |
| DPA1*02:02 | 0.00042 | 0 | 0 | 0 | 0 | 0 | 0.00698 | 0 | 0.00013 | 0 | 0 |
| DPA1*02:03 | 0 | 0 | 0 | 0 | 0 | 0 | 0 | 0 | 0 | 0 | 0 |
| DPA1*03:01 | 0 | 0 | 0 | 0 | 0 | 0 | 0 | 0 | 0 | 0 | 0 |
| DPA1*03:02 | 0 | 0 | 0 | 0 | 0 | 0 | 0 | 0 | 0 | 0 | 0 |
| DPA1*04:01 | 0 | 0 | 0 | 0 | 0 | 0 | 0 | 0 | 0 | 0 | 0 |
| (cont) | DPB1*33:01 | DPB1*34:01 | DPB1*35:01 | DPB1*36:01 | DPB1*39:01 | DPB1*45:01 | DPB1*54:01 | DPB1*72:01 | DPB1*87:01 | DPB1*99:01 |  |
| DPA1*01:03 | 0.00017 | 0.00052 | 0 | 0.00008 | 0.00008 | 0 | 0 | 0.00008 | 0.00008 | 0.00008 |  |
| DPA1*01:04 | 0 | 0 | 0 | 0 | 0 | 0 | 0 | 0 | 0 | 0 |  |
| DPA1*01:05 | 0 | 0 | 0 | 0 | 0 | 0 | 0 | 0 | 0 | 0 |  |
| DPA1*02:01 | 0 | 0.00007 | 0.00034 | 0 | 0 | 0.00025 | 0.00008 | 0 | 0 | 0 |  |
| DPA1*02:02 | 0 | 0 | 0 | 0 | 0 | 0 | 0 | 0 | 0 | 0 |  |
| DPA1*02:03 | 0 | 0 | 0 | 0 | 0 | 0 | 0 | 0 | 0 | 0 |  |
| DPA1*03:01 | 0 | 0 | 0 | 0 | 0.00008 | 0 | 0 | 0 | 0 | 0 |  |
| DPA1*03:02 | 0 | 0 | 0 | 0 | 0 | 0 | 0 | 0 | 0 | 0 |  |
| DPA1*04:01 | 0 | 0 | 0 | 0 | 0 | 0 | 0 | 0 | 0 | 0 |  |
