# Supplementary material for: A combined DPA1∼DPB1 amino acid epitope is the primary unit of selection on the HLA-DP heterodimer
Source: Immunogenetics. 2012 Apr 13;64(8):559–69. doi: 10.1007/s00251-012-0615-3 (PMC3395342; doi:10.1007/s00251-012-0615-3)
Supplement: Supplementary file 2 — (DOC 84 kb) [file 251_2012_615_MOESM2_ESM.doc]

Supplemental Table 2: HLA-DPB1 alleles assigned to either of the three TCE groups according to Zino et al. 2007

**,**

| **DPB1*** | **TCE Group** | **Polymorphism 85-87** | **Linkage DPA1* (%)** |
| --- | --- | --- | --- |
| 09:01 | 1 | EAV | 02:01 (100) |
| 10:01 | 1 | EAV | 02:01 (100) |
| 17:01 | 1 | EAV | 02:01 (100) |
|  |  |  |  |
| 03:01 | 2 | EAV |  |
| 14:01 | 2 | EAV |  |
| 45:01 | 2 | EAV |  |
| 86:01 | 2 | GPM |  |
| 104:01 | 2 | EAV |  |
|  |  |  |  |
| 01:01 | 3 | EAV |  |
| 02:01 | 3 | GPM |  |
| 02:02 | 3 | GPM |  |
| 04:01 | 3 | GPM |  |
| 04:02 | 3 | GPM |  |
| 05:01 | 3 | EAV |  |
| 06:01 | 3 | EAV |  |
| 08:01 | 3 | EAV |  |
| 11:01 | 3 | EAV |  |
| 13:01 | 3 | EAV |  |
| 15:01 | 3 | GPM |  |
| 16:01 | 3 | EAV |  |
| 19:01 | 3 | EAV |  |
| 20:01 | 3 | EAV |  |
| 22:01 | 3 | EAV |  |
| 23:01 | 3 | GPM |  |
| 24:01 | 3 | GPM |  |
| 28:01 | 3 | GPM |  |
| 31:01 | 3 | EAV |  |
| 32:01 | 3 | GPM |  |
| 33:01 | 3 | GPM |  |
| 34:01 | 3 | GPM |  |
| 38:01 | 3 | EAV |  |
| 39:01 | 3 | GPM |  |
| 40:01 | 3 | GPM |  |
| 41:01 | 3 | GPM |  |
| 46:01 | 3 | GPM |  |
| 47:01 | 3 | GPM |  |
| 48:01 | 3 | GPM |  |
| 49:01 | 3 | GPM |  |
| 51:01 | 3 | GPM |  |
| 53:01 | 3 | GPM |  |
| 57:01 | 3 | EAV |  |
| 59:01 | 3 | GPM |  |
| 60:01 | 3 | GPM |  |
| 62:01 | 3 | GPM |  |
| 63:01 | 3 | EAV |  |
| 65:01 | 3 | EAV |  |
| 68:01 | 3 | EAV |  |
| 71:01 | 3 | GPM |  |
| 72:01 | 3 | GPM |  |
| 73:01 | 3 | GPM |  |
| 75:01 | 3 | GPM |  |
| 77:01 | 3 | GPM |  |
| 80:01 | 3 | GPM |  |
| 81:01 | 3 | GPM |  |
| 82:01 | 3 | GPM |  |
| 83:01 | 3 | GPM |  |
| 84:01 | 3 | EAV |  |
| 94:01 | 3 | GPM |  |
| 95:01 | 3 | GPM |  |
| 96:01 | 3 | GPM |  |
| 97:01 | 3 | EAV |  |
| 99:01 | 3 | GPM |  |
| 100:01 | 3 | GPM |  |
| 101:01 | 3 | GPM |  |
| 102:01 | 3 | EAV |  |
| 103:01 | 3 | EAV |  |
| 105:01 | 3 | GPM |  |
| 106:01 | 3 | EAV |  |
| 108:01 | 3 | GPM |  |
| 112:01 | 3 | GPM |  |
| 115:01 | 3 | GPM |  |
| 116:01 | 3 | GPM |  |
